# Traditional Chinese medicine-derived monomers improve bone metabolic imbalance and delay osteoporosis progression by regulating mitochondrial homeostasis

**DOI:** 10.3389/fcell.2026.1846467

**Published:** 2026-06-12

**Authors:** Baohui Wang, Bo Dong

**Affiliations:** Traditional Chinese Medicine Pain Department, TCM Specialty Diagnosis and Treatment Center, Honghui Hospital, Xi’an Jiaotong University, Wuhan, China

**Keywords:** mitochondrial homeostasis, monomers derived from traditional Chinese medicine, osteoblasts, osteoclasts, osteoporosis

## Abstract

Osteoporosis is a systemic metabolic bone disease characterized by reduced bone mass, microarchitectural deterioration, and increased fracture risk. Its pathogenesis is driven by an imbalance between insufficient osteoblast-mediated bone formation and excessive osteoclast-mediated bone resorption. In recent years, mitochondrial homeostasis has emerged as an important pathological hub in this process. Beyond energy production, mitochondria regulate redox balance, mitophagy, apoptosis, senescence, and metabolic adaptation in bone cells. When mitochondrial homeostasis is disrupted, excessive reactive oxygen species accumulation, impaired membrane potential, defective quality control, and metabolic insufficiency collectively suppress osteogenic capacity while promoting osteoclastic activity, ultimately aggravating bone loss. A growing body of evidence suggests that monomers derived from traditional Chinese medicine can partially restore bone metabolic balance by improving mitochondrial function, reducing oxidative stress, and modulating mitochondrial quality control. These compounds have been reported to promote osteoblast survival and differentiation while suppressing osteoclastogenesis and bone resorption. However, the current evidence remains uneven in depth and quality. Most studies are still based on *in vitro* experiments and animal models, whereas direct evidence supporting mitochondria-specific targeting, long-term safety, bioavailability, and clinical applicability remains limited. This review summarizes the pathological role of mitochondrial homeostasis imbalance in osteoporosis and discusses how representative TCM-derived monomers regulate osteogenic and osteoclastic metabolism through mitochondrial mechanisms. It also critically evaluates current limitations and highlights future directions for improving mechanistic rigor and translational value.

## 1 Introduction

Osteoporosis is a common metabolic bone disorder characterized by low bone mass, impaired bone microarchitecture, and increased skeletal fragility. At the biological level, it reflects a chronic imbalance between osteoblast-mediated bone formation and osteoclast-mediated bone resorption. Although anti-resorptive and anabolic agents are already available in clinical practice, their long-term use is still constrained by issues such as safety, durability of efficacy, patient adherence, and limited capacity for precise metabolic regulation (Zhivodernikov et al., 2023; Wu et al., 2020). Increasing evidence indicates that mitochondrial homeostasis is closely involved in the development and progression of osteoporosis. Mitochondria are not only responsible for ATP generation, but also participate in oxidative stress regulation, apoptosis, autophagy, senescence, and lineage differentiation (Yan et al., 2023; Suh and Lee, 2024). In osteoblasts, mitochondrial dysfunction compromises energy supply, redox control, and differentiation programs, thereby weakening bone formation (Ledesma-Colunga et al., 2023; Dobson et al., 2020). In osteoclasts, altered mitochondrial metabolism and excessive oxidative stress can amplify differentiation and resorptive activity, further driving bone loss (Cao G. et al., 2024; Li J. et al., 2023). Therefore, mitochondrial dysregulation is not a secondary bystander event, but a central link connecting impaired osteogenesis with exaggerated osteoclastogenesis.

Against this backdrop, monomers derived from traditional Chinese medicine are gradually emerging as a key focus in the study of osteoporosis mechanisms and drug development due to their multi-target, multi-pathway nature and relatively well-defined mechanisms of action. They have shown significant potential, particularly in regulating oxidative stress, improving mitochondrial function, and restoring the balance of bone metabolism (Moon et al., 2020; Pal et al., 2020). A number of natural compounds have been shown to enhance osteoblast survival and differentiation or suppress osteoclast activation, at least partly through mitochondrial mechanisms (Zhou et al., 2015; Lv et al., 2022). However, this field is still dominated by preclinical evidence, and many published studies remain descriptive rather than mechanistically integrated. In addition, the translational relevance of these compounds is often overstated, given the limited information on pharmacokinetics, bioavailability, tissue delivery efficiency, and long-term safety (Liu et al., 2022; Jing et al., 2019). Accordingly, this review focuses on mitochondrial homeostasis as a shared pathological fulcrum in osteoporosis. We first summarize its role in osteoblast and osteoclast dysfunction, then discuss how representative TCM-derived monomers influence bone metabolism through mitochondrial regulation, and finally highlight the major limitations and future directions of this emerging field.

### 1.1 The pathological role of mitochondrial homeostasis dysregulation in osteoporosis

**FIGURE 1:**
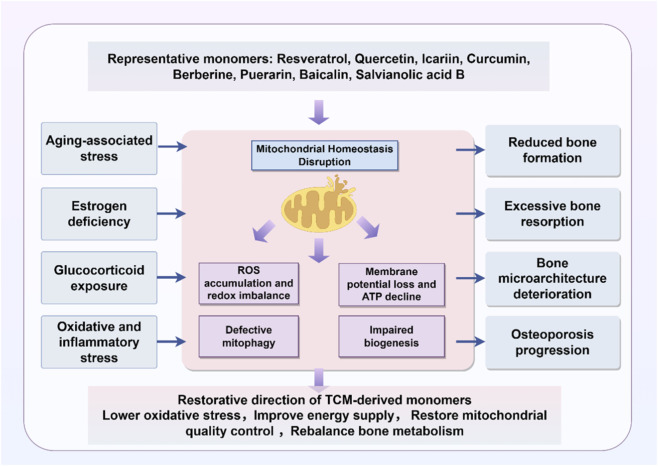
Overall mechanism by which TCM-derived monomers regulate mitochondrial homeostasis in osteoporosis. Age-related stress, estrogen deficiency, glucocorticoid exposure, and oxidative and inflammatory stress can collectively induce mitochondrial homeostasis imbalance, primarily manifested as the accumulation of ROS and redox imbalance, decreased mitochondrial membrane potential and reduced ATP production, impaired mitochondrial autophagy, and insufficient mitochondrial biogenesis. These changes further lead to reduced bone formation, enhanced bone resorption, and disruption of bone microstructure, thereby promoting the progression of osteoporosis. Monomers derived from traditional Chinese medicine, such as resveratrol, quercetin, curcumin, berberine, puerarin, and baicalin, can delay the onset and progression of osteoporosis by alleviating oxidative stress, improving energy supply, restoring mitochondrial quality control, and rebalancing bone metabolism.

Mitochondrial homeostasis dysregulation has been recognized as a key pathological basis for the onset and progression of osteoporosis. Its significance extends beyond energy deficiency, as it simultaneously influences multiple processes, including bone formation, bone resorption, and the remodeling of the bone marrow microenvironment (Liu et al., 2024; Shi et al., 2026). At the mitochondrial level, oxidative phosphorylation, maintenance of the membrane potential, mitochondrial dynamics, mitochondrial biogenesis, and mitochondrial autophagy collectively form a quality control network that ensures the cell’s ability to adapt to metabolic and stress-induced changes (Wang et al., 2020). When this network is compromised, the accumulation of mitochondrial ROS, mitochondrial fragmentation, impaired clearance of damaged mitochondria, and inefficient energy metabolism mutually amplify one another, ultimately shifting bone remodeling from a dynamic equilibrium to a state of persistent imbalance, thereby driving bone loss and increased bone fragility (Riegger et al., 2023; Chen et al., 2025) (Figure 1). In the long term, mitochondrial homeostasis dysregulation is reflected not only in altered bone cell metabolism, but also in impaired bone microarchitecture and increased bone fragility.

For osteoblasts, mitochondrial homeostasis is a key prerequisite for maintaining differentiation, matrix synthesis, and mineralization capacity. Studies have shown that PINK1 deficiency leads to mitochondrial homeostasis abnormalities, decreased membrane potential, and impaired osteogenic differentiation, and exacerbates bone loss in an ovariectomy model, suggesting that defects in mitochondrial quality control directly impair bone formation capacity (Lee et al., 2021; Li W. et al., 2023). Under stress conditions such as mechanical unloading or simulated microgravity, mitochondrial dysregulation and abnormal autophagy further inhibit osteoblast proliferation and differentiation, accompanied by decreased energy status and increased apoptosis. This indicates that mitochondria are not merely passive energy providers for osteogenic differentiation but rather critical regulatory hubs determining cell fate (Ferretti et al., 2024; Michaletti et al., 2017). PGC-1α and APP further support a close link between impaired osteogenesis, mitochondrial dysfunction, and osteoporosis progression by regulating mitochondrial biogenesis, osteoblast differentiation, and oxidative stress (Yu et al., 2018; Pan et al., 2018).

In osteoclasts, mitochondrial homeostasis imbalance does not necessarily result in functional inhibition; on the contrary, in many cases, it leads to an abnormal increase in resorptive activity. Osteoclast differentiation and bone resorption require substantial energy support and rely on ROS as signaling molecules to amplify RANKL-induced downstream processes; therefore, mitochondrial oxidative metabolism and ROS production play a crucial role in their maturation process (Agidigbi and Kim, 2019; Knowles, 2015). Further studies have shown that PGC-1β-mediated mitochondrial biogenesis, iron uptake, and respiratory chain protein assembly form the essential foundation for osteoclast activation and the development of resorptive capacity, while PPARγ-related signaling can amplify this process via PGC-1β, thereby promoting bone loss (Ishii et al., 2009; Wei et al., 2010). Furthermore, mitochondrial biogenesis not only determines osteoclast energy supply but also influences their cytoskeletal organization and resorption function; conversely, abnormalities in NF-κB- and PINK1-mediated mitochondrial quality control can promote excessive osteoclastogenesis through transcriptional regulation and impaired mitochondrial clearance, respectively, further exacerbating the shift toward bone resorption (Zhang et al., 2018; Zeng et al., 2015; Jang et al., 2024).

Overall, the pathological role of mitochondrial homeostasis imbalance in osteoporosis is clearly bidirectional: on the one hand, it impairs osteoblast differentiation, survival, and mineralization through oxidative stress, energy deficiency, and impaired mitochondrial turnover; on the other hand, it amplifies bone loss by promoting osteoclast metabolic reprogramming, mitochondrial biogenesis, and bone resorption processes (Liu et al., 2024; Shi et al., 2026). In other words, mitochondrial abnormalities do not exist solely on the osteogenic or osteoclastic side, but are embedded in both ends of bone remodeling. Through multiple pathways—including autophagy, mitophagy, biogenesis, and inflammatory responses—they link reduced bone formation with enhanced bone resorption into a continuous pathological chain (Wang et al., 2020). For this reason, research focused on mitochondrial homeostasis not only helps elucidate the underlying mechanisms of the dysregulated coupling between osteogenesis and resorption in osteoporosis but also provides a unified theoretical foundation for subsequent discussions on how single compounds derived from traditional Chinese medicine can restore the balance of bone metabolism (Figure 1).

### 1.2 Research progress on monomers derived from traditional Chinese medicine in promoting osteogenic metabolism by regulating mitochondrial homeostasis

The monomers highlighted in this review were selected because they represent natural compounds with relatively concentrated evidence, clearer mechanistic relevance, and stronger representativeness in current studies of bone metabolism. In research on promoting osteogenic metabolism, one of the primary mechanisms that has been repeatedly confirmed is the alleviation of mitochondrial oxidative damage and the restoration of osteoblasts’ stress resistance. Studies on curcumin have shown that it not only alleviates the decline in osteoblast activity and impaired differentiation induced by oxidative stress but also reduces oxidative stress-induced damage to osteoblasts by maintaining mitochondrial function, reducing ROS levels, and stabilizing glycogen synthase kinase 3β (GSK-3β)-related antioxidant signaling (Dai et al., 2017; Li et al., 2020). Evidence supporting quercetin in this regard is also substantial. On one hand, it protects primary human osteoblasts from smoking-related oxidative damage by enhancing antioxidant enzyme activity and maintaining cell viability; on the other hand, it promotes osteogenic differentiation in mesenchymal stem cells and adipose-derived stromal cells, synchronizing the alleviation of oxidative stress with the activation of the osteogenic program (Braun et al., 2011; Li Y. et al., 2015). From a mechanistic perspective, these compounds do not merely act as simple free radical scavengers; rather, by improving the mitochondrial redox state, they create a more stable intracellular environment for osteoblasts to maintain membrane potential, energy supply, and transcriptional programs. Consequently, their effects are more akin to rebuilding the metabolic foundation of osteoblasts, rather than merely transiently increasing the expression of a few osteogenic markers (Bian et al., 2021; Kim et al., 2006) (Figure 1).

In addition to oxidative stress, the mitochondrial-related apoptotic pathway is another key mechanism by which single compounds derived from traditional Chinese medicine promote osteogenesis. Research on puerarin exemplifies this well; it not only inhibits serum deprivation- or glucocorticoid-induced osteoblast apoptosis but also directly intervenes in the mitochondrial-dependent apoptotic process by reducing the Bax/Bcl-2 imbalance, decreasing cytochrome c release, and inhibiting the caspase cascade (Liu et al., 2013; Yu et al., 2015). This suggests that puerarin’s promotion of osteogenic metabolism is not merely a matter of accelerating differentiation, but rather involves first preserving the viability of osteoblasts and then translating this protection into enhanced mineralization and bone formation potential. Further studies indicate that puerarin promotes mesenchymal stem cell proliferation and osteogenic differentiation, enhances new bone formation, and may improve osteogenic dysfunction in inflammatory environments by restoring mitochondrial homeostasis (Yang et al., 2022; Xiang et al., 2025). Therefore, as demonstrated by the example of puerarin, maintaining mitochondrial membrane potential, inhibiting mitochondrial apoptosis, and improving cellular stress survival are, in fact, prerequisites for promoting the restoration of osteogenic metabolism, rather than merely incidental effects.

**FIGURE 2:**
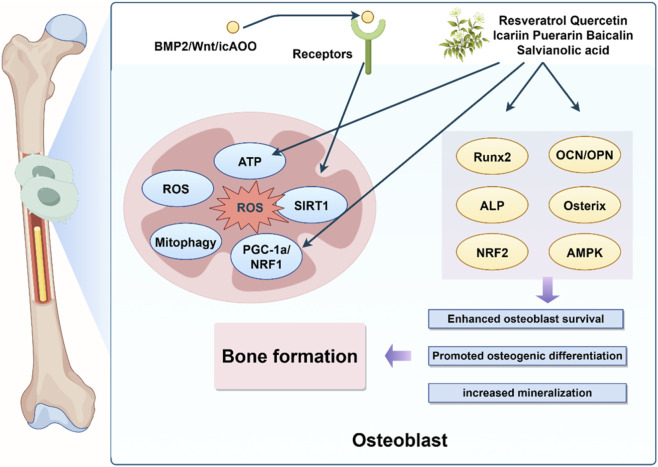
Schematic diagram illustrating how TCM-derived monomers promote osteogenic metabolism through regulation of mitochondrial homeostasis. Upstream stimuli related to BMP2, Wnt, and icariin can influence mitochondrial function and the osteogenic differentiation program in osteoblasts through membrane receptor-mediated signaling. At the mitochondrial level, TCM-derived monomers maintain mitochondrial homeostasis by reducing ROS accumulation, improving ATP production, enhancing SIRT1-mediated protection, promoting mitophagy, and upregulating PGC-1α- and NRF1-dependent mitochondrial biogenesis. Compounds such as resveratrol, quercetin, icariin, puerarin, baicalin, and tanshinic acid further regulate osteogenesis-related molecules and pathways, including Runx2, ALP, NRF2, OCN, OPN, Osterix, and AMPK, thereby enhancing osteoblast survival, promoting osteogenic differentiation and mineralization, and ultimately facilitating bone formation.

When considering the coupling between mitochondrial quality control and osteogenic differentiation, flavonoids and phenolic acid monomers provide more substantial evidence. Baicalin promotes the differentiation of MC3T3-E1 cells into an osteogenic phenotype by activating mTORC1-related osteogenic signaling, while baicalein enhances alkaline phosphatase activity, Runx2 expression, and mineralization levels through the Wnt/β-catenin and MEK/ERK pathways to increase alkaline phosphatase activity, Runx2 expression, and mineralization levels. This suggests that although these monomers may not directly measure mitochondrial dynamics or autophagy in every study, their upstream effects are often consistent with increased mitochondrial energy demand and metabolic remodeling in osteoblasts (Kim JM. et al., 2008; Li SF. et al., 2015; Guo et al., 2011) (Figure 2). Studies on tanshinolic acid B go a step further: it not only promotes osteogenic differentiation of human mesenchymal stem cells but also stimulates osteogenesis and inhibits adipogenesis in a glucocorticoid-induced bone loss model, redirecting cell fate selection toward bone formation via TAZ and MEK-ERK signaling pathways (Xu et al., 2014; Cui et al., 2012; Wang et al., 2019). These findings suggest that the regulation of osteogenic metabolism by TCM-derived monomers is not limited to a single antioxidant effect. Rather, they may exert a long-term osteogenic-promoting effect by improving mitochondrial energy status, promoting the initiation of osteogenic transcriptional programs, and optimizing the balance between osteogenic and adipogenic differentiation. In other words, mitochondrial homeostasis serves here as a comprehensive fulcrum, with TCM-derived monomers simultaneously influencing the three levels of survival, differentiation, and fate selection around this fulcrum (Zhang et al., 2024; Adebayo et al., 2021).

Recent studies have also suggested that certain natural compounds no longer act solely through classical osteogenic signaling pathways, but rather play a more distinct role in mitochondrial damage repair, the restoration of redox homeostasis, and organelle quality control. Naringin can improve bone loss and promote osteogenic differentiation in an ovariectomy model, and it can also upregulate osteogenesis-related genes in bone marrow mesenchymal stem cells; In contrast, naringenin, in a simulated microgravity model, alleviates ROS accumulation, corrects mitochondrial dysfunction, and inhibits osteoblast pyroptosis by activating the NF-κB-related factor 2 and heme oxygenase-1 signaling pathways, demonstrating a more direct role in mitochondrial protection (Guo et al., 2022; Li et al., 2013; Cao S. et al., 2024). Although evidence for berberine is more concentrated in diabetes-related bone defect environments, nanodelivery studies have demonstrated its ability to reduce excessive ROS, improve mitochondrial dysfunction, restore autophagy flux, and enhance osteogenic differentiation capacity (Ming et al., 2024). These findings suggest that the common mechanism by which TCM-derived monomers promote osteogenic metabolism is shifting from merely increasing Runx2, alkaline phosphatase, or mineralized nodules toward the upstream restoration of mitochondrial homeostasis. Overall, existing research in this area has largely established a relatively clear logical chain: TCM-derived monomers first improve mitochondrial oxidative stress, membrane potential, autophagy, and energy metabolism, then enhance the survival, differentiation, and mineralization capacity of osteoblasts or osteoprogenitor cells, ultimately leading to enhanced bone formation. However, it must be acknowledged that the depth of evidence for different compounds varies. Studies directly and simultaneously examining mitochondrial biogenesis, dynamics, autophagy, and osteogenic phenotypes remain scarce, and this is an area that requires further investigation (Yan et al., 2022) (Figure 2).

### 1.3 Recent advances in research on how compounds derived from traditional Chinese medicine inhibit osteoclastic metabolism by regulating mitochondrial homeostasis

**FIGURE 3:**
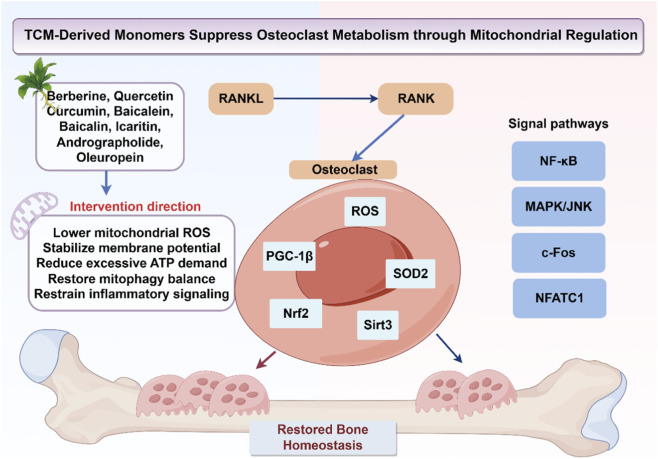
Schematic diagram illustrating how TCM-derived monomers inhibit osteoclastic metabolism and restore bone homeostasis through mitochondrial regulation. RANKL binding to RANK activates osteoclast-related signaling pathways, thereby promoting osteoclast differentiation and bone resorption. During this process, increased mitochondrial ROS, unstable membrane potential, elevated energy demand, and disrupted mitochondrial quality control collectively contribute to abnormal osteoclast activation. TCM-derived monomers, including resveratrol, quercetin, curcumin, baicalin, herba epimedii extract, andrographolide, and oleuropein, can reduce mitochondrial ROS, stabilize mitochondrial membrane potential, alleviate excessive metabolic stress, restore mitophagy balance, and suppress inflammatory signal amplification. At the same time, they inhibit pathways such as PGC-1β, NF-κB, MAPK/JNK, c-Fos, and NFATc1, while modulating Nrf2-, SOD2-, and Sirt3-related mitochondrial regulation, ultimately attenuating osteoclast differentiation and bone resorption and promoting the restoration of bone homeostasis.

In addition to promoting osteogenic metabolism, suppression of osteoclastic metabolism is equally essential for restoring bone remodeling balance in osteoporosis. Unlike osteoblasts, osteoclast differentiation and bone resorption are more directly dependent on mitochondrial metabolism. RANKL-induced osteoclastogenesis not only requires a continuous energy supply but is also accompanied by increased mitochondrial ROS, changes in membrane potential, and amplification of mitochondrial-related signaling. These changes further drive the activation of key transcriptional programs such as c-Fos and NFATc1, ultimately leading to the formation of mature osteoclasts and enhanced bone resorption (Srinivasan et al., 2010; Udagawa et al., 2021). Previous studies have shown that mitochondrial ROS are not merely metabolic byproducts but important signaling molecules involved in the regulation of osteoclast differentiation; when mitochondrial antioxidant regulatory systems such as SOD2 and Sirt3 are impaired, the accumulation of mitochondrial ROS is significantly enhanced, further promoting osteoclastogenesis and bone resorption activity (Agidigbi and Kim, 2019; Kim et al., 2017). This suggests that inhibiting osteoclastic metabolism by targeting mitochondrial homeostasis is not a peripheral regulation but rather acts directly on the metabolic core of osteoclasts. Recent studies have further suggested that Nrf2 exerts a significant inhibitory effect on osteoclastogenesis; its protective role lies not only in reducing cytoplasmic oxidative stress but also in suppressing the excessive production of mitochondrial ROS. Therefore, limiting osteoclast differentiation by regulating mitochondrial redox balance has become a crucial theoretical foundation for understanding the anti-bone resorption effects of single compounds derived from traditional Chinese medicine (Yang et al., 2023) (Figure 3).

Among monomers derived from traditional Chinese medicine, studies on berberine and quercetin best illustrate this mechanism of action. Berberine sulfate significantly inhibits RANKL-induced osteoclast differentiation and downregulates NF-κB and NFAT-related pathways, indicating that it can block downstream bone resorption signals early in osteoclast differentiation (Zhou et al., 2015). Subsequent studies further revealed that berberine can also inhibit the expression of c-Fos and NFATc1, thereby reducing the formation of TRAP-positive osteoclasts and their bone resorption function; this effect is closely related to its reduction of oxidative stress and inhibition of inflammatory signal amplification (Han and Kim, 2019). Quercetin is another representative monomer; classic studies have shown that quercetin can inhibit RANKL-induced osteoclast differentiation and reduce the formation of mature osteoclasts by interfering with the activation processes of NF-κB and AP-1 (Wattel et al., 2004). These findings suggest that while certain natural monomers appear to inhibit transcriptional signaling on the surface, their underlying mechanisms are often consistent with reduced mitochondrial ROS, alleviated oxidative stress, and attenuated osteoclastic metabolic processes. Thus, TCM-derived monomers inhibit osteoclastic metabolism not merely by blocking inflammatory factors, but by restoring mitochondrial homeostasis and reducing excessive osteoclast activation. Curcumin supports this view by suppressing osteoclastogenesis through reduced RANKL expression and inhibition of RANKL-related signaling and autophagy in osteoclast precursors (Oh et al., 2008).

Scutellaria and tea polyphenol monomers further demonstrate that inhibiting osteoclastic metabolism does not simply involve suppressing cellular activity, but rather involves correcting an imbalance in the coupling between mitochondrial quality control and differentiation programs. Baicalin can inhibit osteoclast differentiation and induce apoptosis in mature osteoclasts, suggesting that it not only reduces osteoclast formation but also shortens the survival time of established osteoclasts (Kim MH. et al., 2008). Scutellarin, on the other hand, exhibits a concentration-dependent bidirectional effect: at high concentrations, it inhibits osteoclast differentiation and bone resorption (Figure 3). This further suggests that the regulation of osteoclastic metabolism by natural monomers is not a linear relationship but is closely related to dose, metabolic background, and cellular state (Lu et al., 2018). There is a wealth of evidence regarding epigallocatechin gallate (EGCG). Some studies indicate that it can inhibit osteoclast formation by downregulating NFATc1 and osteoclast-related gene expression, while others show that it can alleviate inflammation-associated bone resorption and experimental bone destruction (Dong et al., 2022; Morinobu et al., 2008). More recent work has also found that this type of polyphenol can persistently inhibit the expression of osteoclast differentiation markers in primary bone marrow cells and RAW264.7 cells, suggesting that its effects are not limited to a single model (Xu et al., 2019). Taken together, these studies suggest that when inhibiting osteoclastic metabolism, TCM-derived monomers likely maintain mitochondrial quality control balance by reducing mitochondrial ROS, alleviating mitochondrial stress, and limiting abnormal autophagy activity—in addition to the classic NF-κB, MAPK, and NFATc1 pathways—thereby making it difficult for osteoclasts to sustain a state of high bone resorption.

Recent studies have further advanced this line of research to a more clinically relevant level. Icariin has been shown to attenuate RANKL-mediated osteoclastogenesis and reduce the increase in mitochondrial mass and function required for osteoclast differentiation, suggesting that it does not merely inhibit osteoclastogenesis at the receptor signaling level, but directly influences the metabolic remodeling process of osteoclasts (Huang et al., 2023). More recent studies have also shown that icariin can inhibit the miR503/RANK axis through ESR1-related mechanisms, thereby reducing osteoclast differentiation and bone loss (Xie et al., 2025). Probenecid exhibits a distinct anti-resorptive effect by inhibiting JNK, reducing ROS production, and downregulating COX-2; this finding further supports the importance of reducing oxidative stress and inflammatory amplification in inhibiting resorptive metabolism (Cheng and Kim, 2020). Oleuropein and its derivatives have also been reported to negatively regulate the expression of resorption-related genes, thereby reducing osteoclast function (Rosillo et al., 2020) (Figure 3). Furthermore, andrographolide and baicalin have also demonstrated potent anti-resorptive effects; the former reduces bone resorption by inhibiting NF-κB and ERK/MAPK signaling, while the latter inhibits osteoclast formation and mitigates bone loss in an ovariectomy model (Zhai et al., 2014; Ma et al., 2022). Overall, the common mechanism by which TCM-derived monomers inhibit osteoclastic metabolism through the regulation of mitochondrial homeostasis is now relatively clear: first, they reduce mitochondrial ROS and abnormal metabolic stress; second, they limit osteoclast differentiation, maturation, and bone resorption; and finally, they result in reduced bone loss. However, studies directly and simultaneously assessing mitochondrial dynamics, membrane potential, autophagy, and osteoclastic phenotypes remain scarce, which is a gap that needs to be addressed for further mechanistic elucidation. Promotion of osteogenic metabolism and inhibition of osteoclastic metabolism are complementary processes in the maintenance of bone remodeling balance. Therefore, in addition to enhancing osteogenesis, suppression of osteoclastic metabolism is also essential for the anti-osteoporotic effects of TCM-derived monomers.

### 1.4 Common mechanisms and distinctive features of how single compounds derived from traditional Chinese medicine regulate mitochondrial metabolism in osteoblasts and osteoclasts

Overall, the regulation of osteoblasts and osteoclasts by TCM-derived compounds does not follow two separate pathways but rather revolves around the common pivot of mitochondrial homeostasis. At the mitochondrial level, both osteoblasts and osteoclasts rely on a continuous energy supply, ROS control, and quality control networks to maintain their functions; however, their requirements for mitochondrial metabolism differ. Osteoblasts rely more on a stable energy supply and moderate mitochondrial biogenesis to support differentiation, matrix synthesis, and mineralization; osteoclasts, in contrast, rely more on higher levels of oxidative metabolism and mitochondrial signal amplification to maintain differentiation, maturation, and bone resorption activity (Lee et al., 2017; Sautchuk and Eliseev, 2022). Therefore, although many monomers derived from traditional Chinese medicine appear to simultaneously promote osteogenesis and inhibit osteoclastogenesis, their mechanism does not involve uniformly enhancing mitochondrial activity in both cell types. Instead, they correct the respective abnormal metabolic states of each: osteoblasts recover from an inefficient, damaged mitochondrial environment, while osteoclasts return from an overactivated, hypermetabolic state (Xu et al., 2023; Pan et al., 2024). This may explain why the same natural monomers often enhance osteogenic markers while suppressing osteoclast-related molecules, as they target the shared mitochondrial homeostasis network underlying bone remodeling, although its manifestations differ between the 2 cell types (Figure 3).

From the perspective of common mechanisms, the restoration of redox balance may be the most fundamental and widespread mode of action for monomers derived from traditional Chinese medicine. In both osteoblasts and osteoclasts, abnormal accumulation of ROS in mitochondria disrupts normal metabolic processes: in the former, this manifests as impaired differentiation, reduced mineralization, and accelerated aging; in the latter, it manifests as enhanced differentiation, increased bone resorption, and amplified inflammatory signaling (Gao et al., 2018; Ling et al., 2021). In this context, nuclear factor erythroid 2-related factor 2 (Nrf2) and its downstream antioxidant pathways play a bidirectional regulatory role. On the one hand, moderate activation of Nrf2 helps maintain the survival and differentiation environment of osteoblasts; on the other hand, upregulation of Nrf2 can limit the amplification of ROS signaling in osteoclasts, thereby inhibiting bone resorption-related processes (Sun et al., 2015; Han et al., 2022). Recent studies also suggest that Nrf2’s regulation of osteogenesis and osteoclastogenesis is not entirely symmetrical but exhibits distinct cell-type-specific differences. This implies that when TCM-derived monomers exert their beneficial effects by modulating oxidative stress, they do not simply reduce ROS levels indiscriminately in all bone cells; rather, they likely maintain ROS within a range that promotes bone formation without excessively stimulating bone resorption (Wang et al., 2023). Therefore, from an integrative mechanistic perspective, the regulation of mitochondrial ROS and the antioxidant network by TCM-derived monomers constitutes the primary common foundation for their simultaneous promotion of osteogenesis and inhibition of osteoclastogenesis.

In addition to redox balance, AMP-activated protein kinase (AMPK)-mediated energy sensing and autophagy regulation constitute another key common pathway. In osteoblasts, AMPK activation is typically associated with improved energy homeostasis, enhanced autophagy, and restored differentiation capacity, suggesting that osteoblasts require a metabolic environment capable of maintaining a balance between energy supply and organelle renewal (Kanazawa et al., 2018; Li et al., 2018). In osteoclasts, however, AMPK primarily acts to restrict excessive differentiation and high-energy resorption states; AMPKα1 deficiency promotes osteoclastogenesis and exacerbates bone loss, while AMPK inhibition weakens the inhibitory effect of bone-protective signals on osteoclasts (Ribeiro et al., 2023; Tong et al., 2020). Autophagy and mitophagy, which are closely associated with AMPK, similarly exhibit these characteristics of coexisting similarities and differences. Overall, moderate autophagy helps clear damaged mitochondria and maintain cellular metabolic homeostasis, serving as a crucial condition for osteoblasts to maintain their function; in osteoclasts, however, autophagy and mitophagy are more involved in their differentiation, fusion, and the maintenance of bone resorption functions. Therefore, it is not simply a case of “the stronger, the better,” but rather a matter of maintaining a dynamic balance (Wang et al., 2020; Zeng et al., 2022; Kubat et al., 2023; Fan et al., 2025). This also suggests that when TCM-derived compounds exert their effects by regulating mitochondrial quality control, their action cannot be simply categorized as promoting or inhibiting autophagy; rather, it should be understood as restoring more appropriate mitochondrial renewal rhythms and energy allocation patterns tailored to the pathological states of different bone cells.

As a distinctive feature, given their distinct characteristics and the overall balance of bone remodeling, the fundamental differences in mitochondrial metabolism between osteoblasts and osteoclasts mean that the ultimate effects of single-component TCM extracts cannot be understood solely in terms of a single cell type (Buccoliero et al., 2021). Peroxisome proliferator-activated receptor γ coactivator 1α (PGC-1α) is more inclined to support osteogenic lineage commitment, mitochondrial biogenesis, and the maintenance of bone formation, whereas mitochondrial remodeling associated with the high resorption state of osteocytes manifests more as a distinct metabolic amplification program (Albert et al., 2022). Consequently, even when the same compound appears to improve mitochondrial homeostasis, its primary effect in osteoblasts is often to restore differentiation, survival, and mineralization, whereas in osteoclasts, it primarily suppresses differentiation, maturation, and the intensity of bone resorption. Furthermore, the true value of TCM-derived monomers lies not in selectively enhancing or suppressing either the osteogenic or osteoclastic pathways, but in restoring bone remodeling to a relatively balanced state by simultaneously regulating the mitochondrial status of both cell types (Kim et al., 2020; Zhang et al., 2020; Hung et al., 2020). This point also distinguishes this review from traditional reviews on osteoporosis, as it no longer treats the promotion of osteogenesis and the inhibition of osteoclast activity as two parallel outcomes, but rather views them as bidirectional remodeling effects arising from the common pathological fulcrum of mitochondrial homeostasis. Future studies should move beyond single-cell regulation and further investigate how mitochondrial homeostasis influences the bone marrow microenvironment and intercellular communication during bone remodeling.

## 2 Summary and outlook

Mitochondrial homeostasis imbalance is increasingly recognized as a central pathological driver of osteoporosis rather than a secondary metabolic consequence. By linking oxidative stress, defective quality control, impaired osteoblast function, and excessive osteoclast activation, it provides a useful framework for understanding how bone remodeling shifts toward persistent bone loss. In this context, monomers derived from traditional Chinese medicine have shown promising regulatory potential. Many of these compounds appear capable of improving osteogenic metabolism and restraining osteoclastic activity by reducing mitochondrial oxidative stress, stabilizing membrane potential, modulating mitophagy and apoptosis, and supporting metabolic adaptation in bone cells.

At the same time, the current evidence should be interpreted with caution. First, most available studies remain limited to cell experiments and animal models, and the strength of evidence varies substantially among compounds. Second, many reports describe improvements in ROS levels, autophagy markers, or osteogenic and osteoclastic phenotypes, but do not sufficiently establish a direct causal relationship between mitochondrial homeostasis and the observed bone-protective effects. Third, comparative data between different monomers are scarce, making it difficult to define their relative potency, preferred targets, or most suitable pathological settings. Finally, key translational issues, including pharmacokinetics, oral bioavailability, formulation stability, bone-targeted delivery, and long-term safety, are still insufficiently addressed.

Future research should move beyond descriptive observations and place greater emphasis on mechanistic depth and translational validity. More rigorous studies are needed to verify mitochondria-specific targets, clarify cell-type-specific regulatory networks in osteoblasts and osteoclasts, and integrate mitochondrial findings with the broader bone marrow microenvironment. Multi-omics approaches, standardized compound preparation, and optimized delivery systems may further improve reproducibility and clinical relevance. Rather than viewing TCM-derived monomers simply as general antioxidants or natural anti-resorptive agents, their value should be reassessed from the perspective of mitochondrial remodeling and coordinated restoration of bone metabolic balance. With better mechanistic validation and stronger translational design, these compounds may become meaningful candidates for future osteoporosis intervention strategies.

## Glossary

OP: Osteoporosis
TCM: Traditional Chinese medicine
ROS: Reactive oxygen species
ATP: Adenosine triphosphate
ΔΨm: Mitochondrial membrane potential
AMPK: AMP-activated protein kinase
Nrf2: Nuclear factor erythroid 2-related factor 2
SIRT1: Sirtuin 1
SIRT3: Sirtuin 3
PGC-1α: Peroxisome proliferator-activated receptor gamma coactivator 1-alpha
NRF1: Nuclear respiratory factor 1
TFAM: Mitochondrial transcription factor A
PINK1: PTEN-induced kinase 1
Parkin: Parkin RBR E3 ubiquitin protein ligase
BMP2: Bone morphogenetic protein 2
IGF-1: Insulin-like growth factor 1
ALP: Alkaline phosphatase
OCN: Osteocalcin
OPN: Osteopontin
RANKL: Receptor activator of nuclear factor κB ligand
RANK: Receptor activator of nuclear factor κB
NF-κB: Nuclear factor κB
MAPK: Mitogen-activated protein kinase
JNK: c-Jun N-terminal kinase
NFATc1: Nuclear factor of activated T cells 1
TRAP: Tartrate-resistant acid phosphatase
CTSK: Cathepsin K
ACP5: Acid phosphatase 5
DC-STAMP: Dendritic cell-specific transmembrane protein
ATP6V0D2: ATPase H+ transporting V0 subunit d2
SOD2: Superoxide dismutase
